# Epidemiology, Virulence Factors, and Antibacterial Resistance of *Klebsiella* spp.: The Known Unknowns

**DOI:** 10.3390/medicina62030546

**Published:** 2026-03-15

**Authors:** Angelika Krūmiņa, Indra Zeltiņa, Lauma Māra Vikmane, Aigars Reinis, Ludmila Vīksna

**Affiliations:** 1 Department of Infectology, Riga Stradins University, 1007 Riga, Latvia; indra.zeltina@rsu.lv (I.Z.); ludmila.viksna@rsu.lv (L.V.); 2 Riga East Clinical University Hospital, 1079 Riga, Latvia; 3 Faculty of Medicine, Riga Stradins University, 1007 Riga, Latvia; lauma.vikmane@rsu.edu.lv; 4 Department of Biology and Microbiology, Riga Stradins University, 1007 Riga, Latvia; aigars.reinis@rsu.lv; 5 Pauls Stradins Clinical University Hospital, 1002 Riga, Latvia

**Keywords:** *Klebsiella* spp., *K. pneumoniae*, epidemiology, virulence factors, antibacterial resistance

## Abstract

*Klebsiella* spp. are among the most prominent bacteria in nature and can be found in different environments, including soil, vegetation, and surface waters. The most important species of the *Klebsiella* genus is *Klebsiella pneumoniae*, which is recognized as an alarming opportunistic pathogen responsible for approximately 70% of human infections within their family. Due to their evolving hypervirulence and antimicrobial resistance, there is an increasing amount of research about the trends and mechanisms of virulence factors and epidemiology. Understanding pathogenetic mechanisms is important to advance diagnostic methods and treatment. In this review, we aim to summarize the available information and recent development in research of *Klebsiella* spp., specifically focusing on the trends in epidemiology, virulence factors, and the development of antimicrobial resistance.

## 1. Introduction

*Klebsiella* spp. are Gram-negative, non-motile, facultative anaerobic bacilli of the *Enterobacteriaceae* family, characterized by a polysaccharide capsule and widespread presence in nature. In humans, they commonly colonize the skin, oropharynx, and gastrointestinal tract as commensals [1].

**Figure 1:**
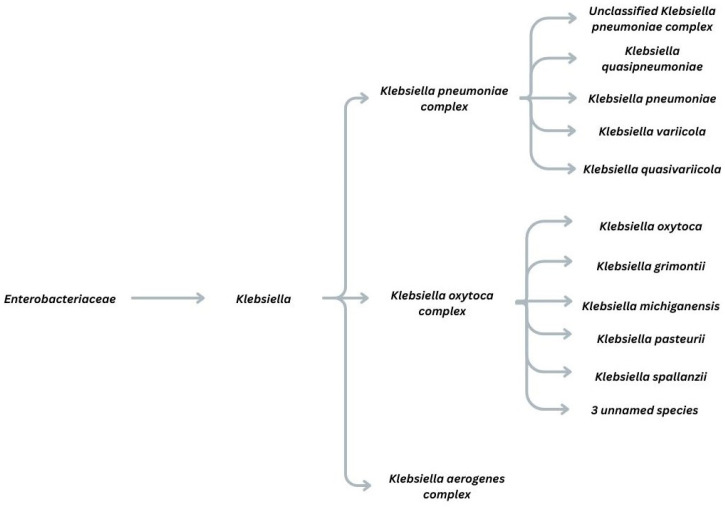
Taxonomic levels of the *Klebsiella* species.

The *Enterobacteriaceae* family encompasses a large order of Gram-negative bacteria (*Klebsiella*, *Escherichia*, *Enterobacter*, *Serratia*). The genus is then further divided into complexes, which are clusters of distinct but genetically similar species that are often difficult to differentiate through traditional biochemical or phenotypic methods. This is portrayed in Figure 1. Clinically important complexes from the *Klebsiella* genus include the *Klebsiella pneumoniae* species complex (KpSC), the *Klebsiella oxytoca* complex, and the *Klebsiella aerogenes* complex. KpSC is divided into seven pathotypes (Kp1–Kp7). *K. pneumoniae sensu stricto* (Kp1, *K. pneumoniae*) is the most common clinical pathogen, accounting for about 70% of human *Klebsiella* infections, causing pathologies such as pneumonia, bacteremia, meningitis, and liver abscess [1,2,3]. Other members, including *K. quasipneumoniae* and *K. variicola*, are frequently misidentified as *K. pneumoniae* because of high genetic similarity [3]. The *Klebsiella oxytoca* species complex (KoSC) was once considered a single species but now comprises nine species, including *K. grimontii*, *K. michiganensis*, *K. pasteurii*, and *K. spallanzanii. K. oxytoca* is typically linked to antibiotic-associated hemorrhagic colitis (AAHC): after antibiotic therapy disrupts normal gut flora, cytotoxic *K. oxytoca* overgrows. This complex is distinguished from other *Klebsiella* by the intrinsic, chromosomally encoded β-lactamase *blaOXY*, which serves as a molecular marker for its classification [4]. Bacteria of the *Klebsiella aerogenes* complex (formerly *Enterobacter aerogenes*), comprising three species and two subspecies, are typically motile and have a distinct flagellar chemotaxis region, unlike most other *Klebsiella*. It also carries an intrinsic, chromosomal *ampC* gene encoding a class C β-lactamase, whereas other *Klebsiella* species usually have class A β-lactamases. These two traits made *K. aerogenes* appear more like *Enterobacter* than *Klebsiella*. However, genetic sequencing later showed that its genome aligns with the *Klebsiella* cluster, and detailed genomic analysis confirmed a closer phylogenetic relationship to KpSC than to any *Enterobacter* species. It is a common cause of nosocomial infections and carries intrinsic resistance determinants that enable the rapid development of resistance during third-generation cephalosporin treatment, often necessitating escalation of therapy [5].

Virulence is the ability of a microbe to cause disease by damaging the host [6]. Historically, *K. pneumoniae* strains were classified as classical (cKp), typically opportunistic in immunocompromised patients, and hypervirulent (hvKp), which could infect healthy individuals and cause severe community-acquired disease. Classical strains were strongly associated with multidrug resistance (MDR) and the broader antimicrobial resistance (AMR) crisis, whereas hvKp remained largely antibiotic-susceptible [7]. Over the last decade, evolutionary changes have driven the emergence and spread of strains that are both hypervirulent and antimicrobial-resistant, increasingly replacing less virulent, antibiotic-susceptible strains [8].

Antimicrobial resistance (AMR) is the ability of microorganisms to withstand antibacterial, antiviral, antiparasitic, and antifungal agents [9]. AMR pathogens are increasing, limiting treatment options. *K. pneumoniae* alone causes 20% of global AMR-related deaths, making it a “critical priority” pathogen [10,11]. Numerous studies report rising AMR in *Klebsiella* spp. For instance, a longitudinal multicenter study in China showed carbapenem resistance increasing from 2.9% in 2005 to 30% in 2023, despite its role as a last-resort antibiotic. In some cohorts, resistance to amikacin and gentamicin exceeds 90% in specific ST11-K64 lineages [12]. In a Polish hospital, ESBL-producing *K. pneumoniae* rose from 30% in 2017 to 51% in 2021, surpassing susceptible strains [13]. In Iran, 84.6–94.8% of *K. pneumoniae* bloodstream isolates are multidrug-resistant (MDR), with many classified as extensively drug-resistant (XDR) or pandrug-resistant (PDR) [14]. These data indicate the global spread of AMR and growing numbers of MDR phenotypes.

Epidemiology tracks the distribution, transmission, and risk factors of pathogens [6]. Key current trends include the global dissemination of high-risk clones [15], the convergence of resistance and hypervirulence in *K. pneumoniae* [16], and, under the One Health framework, the role of environmental reservoirs as hotspots for horizontal gene transfer [17]. Understanding the evolution of MDR strains and the genetic basis of virulence is crucial for developing new diagnostics and therapies. Accordingly, we analyze the global epidemiological shift in high-risk clones and summarize the mechanisms of genetic convergence leading to these strains.

## 2. Epidemiology

### 2.1. Klebsiella pneumoniae Complex

*Klebsiella* spp. show shifting epidemiology: carbapenem-resistant *K. pneumoniae* (crKp), once mainly healthcare-associated, and hypervirulent *K. pneumoniae* (hvKp), once largely community-acquired in the Asian Pacific Rim, now increasingly overlap. High genetic plasticity enables strains to acquire and combine MDR and hypervirulence via plasmid exchange, recombination/fusion, immune deficits, and metabolic shifts. Plasmids are the main drivers of hypervirulence and resistance spread. During the horizontal transfer of large conjugative plasmids, hvKp can acquire resistance plasmids (e.g., *blaKPC-2*, *blaNDM-1*) to form CR-hvKp, and MDR crKp can acquire virulence plasmids (pLVPK, pk2044) to form hv-crKp, producing new high-risk “superbug” clones [7,15,18,19]. Recombination, often mediated by insertion sequences (ISs), generates hybrid plasmids carrying both virulence and resistance genes [19,20]. Formerly mainly plasmid-borne, hypervirulence factors now also appear as virulence plasmid fragments (*rmpA2*, *iucABCD*) integrated into the chromosome via ISs such as IS26 and ISKpn1 [21,22], ensuring stable inheritance without the metabolic burden of large plasmids. High-risk clones often lack functional-type I-E CRISPR-Cas and restriction–modification systems, which normally block foreign DNA [7], making them highly permissive to MDR and virulence plasmid acquisition [8].

Key high-risk clones (hypervirulent and resistant) driving global spread belong to Clonal Group 258, including ST258, ST11, and ST512, the most widespread MDR group [7]. ST258 and ST512 are endemic in the United States and Southern Europe, while ST11 is the dominant carbapenem-resistant clone in China [1]. Newer clones such as ST147, ST101, and ST307 have also emerged and can outcompete established lineages in some hospitals [23]. In China, a longitudinal study of 1017 isolates showed hv-crKp ST11 prevalence increasing from 28.2% in 2016 to 45.7% in 2020 [20]. Additionally, molecular data from 2018 to 2023 indicate that CR-hvKp ST23 in China has an average annual growth rate of 30–59% [24]. Core genome analysis of >5000 Chinese isolates found that CR-hvKp now represents 44.6% of all carbapenem-resistant and 69.5% of all hypervirulent isolates, demonstrating rapid expansion of convergent strains [25]. ST23 has been reported in all six WHO regions. Additionally, other sequence types have spread across large areas of the world, as seen in Table 1, highlighting the global spread of *K. penumoniae*. Given that *K. pneumoniae* causes 20–30% of nosocomial pneumonias worldwide and is a leading cause of hospital-acquired Gram-negative bacteremia, the WHO lists carbapenem-resistant *K. pneumoniae* as a “critical priority” pathogen and a major global cause of healthcare-associated infections [11].

*K. variicola* and *K. quasipneumoniae* are non-pneumoniae members of KpSC that cause about 10–20% of KpSC-associated disease [26]. Once considered less virulent, these species are increasingly acquiring high-risk plasmids. *K. variicola* isolates carrying carbapenemases (e.g., *blaKPC-2*, *blaNDM*) and virulence factors have been reported, including a hypervirulent MDR ST771 outbreak in neonatal units in Bangladesh. In some regions, *K. quasipneumoniae* acts as a reservoir for *blaOXA-23* and *blaNDM-1*, conferring β-lactam resistance [3,21]. The WHO notes that, while hypervirulent ST23 *K. pneumoniae* is a global priority, the detection of hypervirulence in other species is still limited in routine laboratories, likely underestimating their prevalence and risk [11].

### 2.2. Klebsiella oxytoca Complex

Species other than *K. pneumoniae* also show rising virulence and antimicrobial resistance. Clinical reports of the KoSC are most frequent in North America, Western Europe, and the Asia–Western Pacific region. *K. oxytoca and K. michiganensis* are the primary species of this complex associated with extraintestinal infections, though all nine species can cause disease. Outbreaks are usually associated with contaminated hospital water sources in high-risk settings like NICUs and oncology wards. All members of this complex carry intrinsic chromosomal *blaOXY* genes, which create resistance to penicillin-based antibacterial agents, as well as *fosA* (fosfomycin resistance), and in most species *oqxAB* (quinolone resistance) [4]. An important trend in the evolution of the KoSC is its ability to access the same plasmid reservoirs as *K. pneumoniae*, which allows the KoSC lineages to rapidly evolve through horizontal gene transfer, acquiring carbapenemases and colistin resistance determinants [27]. Due to the emergence of plasmid-mediated *tet(X4)* genes, the whole genus has the ability to develop high-level tigecycline resistance [17]. SENTRY data (2013–2019) indicate that carbapenem non-susceptibility in the KoSC remained low overall (1.8%) but increased over time. Regional differences are marked: resistance reached 6.4% in China and 6.1% in Thailand, versus 0.9% in North America.

Within KoSC, clonal complex 2 (ST2, ST199, ST176) is emerging as a high-risk lineage, frequently carbapenem-resistant and rapidly disseminating in regions worldwide [2,4]. ST2 is associated with rapid dissemination via clonal expansion in Germany, the UK, and Ireland, and it frequently presents as an MDR phenotype. ST199 is the dominant strain in the US and is frequently associated with colistin resistance genes, such as *mcr9.1* and *mcr9.2*. Lastly, the ST176 lineage is globally disseminated (Australia, Sweden, the UK), and, similarly to other high-risk lineages, it often possesses gene mutations (*gyrAS83T*) conferring quinolone resistance [4,28].

### 2.3. Klebsiella aerogenes Complex

The *K. aerogenes* complex (KAC) is an important cause of hospital-acquired infections, particularly in ICUs, causing a wide range of nosocomial infections, including urinary tract, pulmonary, wound, peritoneal, meningeal, and bloodstream infections [5]. Mortality due to KAC ranges from 13.5% to 28%. KAC exhibits remarkable clonal diversity, represented by over 366 ST and 214 clonal cluster. There are multiple globally disseminated clusters with carbapenemase-encoding genes (ST93 of clone cluster (CC) 1, ST4 of CC2, ST93 of CC3) determined in multiple continents like North America, Europe, Asia, and Oceania. All members of KAC typically carry the intrinsic chromosomal *ampC* gene, which confers resistance to most β-lactams when overproduced. In addition to the chromosomal *ampC* gene, other resistance determinants carried on plasmids are frequently present. Currently, approximately 18.8% of KAC genomes deposited in public databases contain acquired carbapenemase genes, predominantly *KPC-2* and *NDM-1*. Similarly to KpSC, there is a trend of MDR and hypervirulent markers converging within the same lineage. For example, there is evidence of KAC strains acquiring large hybrid plasmids carrying both hypervirulent markers and carbapenemases genes, portrayed by the ST432 isolate in the USA. This strain carried the complete aerobactin operon (*iucABCD* and *iutA*) and the *rmpA2* gene, which regulate hypermucoviscosity, indicating hypervirulent strains and resistance genes *blaNDM5* and *blaCTX-M-5* conferring antibacterial resistance. This suggests the emergence of convergent strains in KAC and dangerous interspecies transfer of genetic material, contributing to the creation of more hypervirulent and resistant strains [5].

### 2.4. One Health

Epidemiological trends promote gene transfer and clonal replacement through “transmission hubs”, as illustrated by the One Health perspective. Multiple sources report that pathogens of *K. pneumoniae*, *K. oxytoca*, and *K. aerogenes* complexes are widespread in nature, with environmental and animal reservoirs (e.g., urban/hospital wastewater, poultry, livestock) serving as hubs for horizontal transfer of resistance genes [4,5,17]. *K. pneumoniae*’s marked genetic plasticity allows it to thrive in varied natural and clinical settings [6], exposing it to antibiotics from human wastewater, pharmaceutical runoff, and agriculture, and enabling resistance development outside hospitals [17]. Regarding KoSC, a major focal point for the emergence of high-risk KoSC strains is in their association with swine. The majority of *tet(X4)* resistance gene carrying isolates were livestock-associated, and of those, 17% possessed hypervirulence marker *iuc3*, largely isolated from swine [17]. *K. pneumoniae*, as a common commensal in humans and animals, cycles between hosts, increasing contact with hypervirulent or MDR strains, promoting plasmid exchange and evolutionary convergence [1,8]. Extensive interaction opportunities allow strains carrying more virulence and resistance plasmids to outcompete those with fewer, driving “clonal replacement”. For example, the ST11-KL64 subclone systematically replaced ST11-KL47 due to its broader virulence and resistance plasmid repertoire and resulting fitness advantage [17]. Additionally, an increasing number of convergent strains of *K. pneumoniae* carrying high-risk plasmids increases the likelihood of interspecies plasmid transference, as per the previously mentioned example of ST432 KAC strain [5]. Documented examples include *K. quasipneumoniae* ST367 with KpVP-1 virulence plasmids and *K. variicola* ST595 co-harboring KPC-2 [5,26].

Currently, global risk is considered to be “moderate” because of gaps in surveillance and molecular testing [11]. Existing CR-hvKp infections, such as meningitis and necrotizing fasciitis, already carry mortality rates of 25–42% [15]. Projections are far worse: by 2030, >50% of invasive *K. pneumoniae* may be resistant to carbapenems and third-generation cephalosporins [29]. If plasmid-mediated convergence continues, the next generation of *K. pneumoniae* could become resistant to all available antimicrobials, including last-resort agents like colistin and Ceftazidime–avibactam (CZA), leading to a “clinical crisis” [30]. Total annual deaths from antibiotic-resistant bacteria, including *Klebsiella*, may reach 10 million by 2050 [13].

## 3. Virulence Factors

Virulence factors are the products of the multifactorial synergy of different genetic elements that determine capsular production, siderophore-mediated iron acquisition, and specialized regulatory systems.

### 3.1. Adhesion Factors

The ability of *Klebsiella* spp. to adhere to host tissues and medical devices is mediated by fimbriae (pili). Type 1 fimbriae and Type 3 fimbriae (*mrk*) promote adhesion to epithelial cells and abiotic surfaces. Type 3 fimbriae are particularly important for robust biofilm formation. Biofilm production is often regulated by the mrkH transcriptional activator and the QseBC signaling system [1,4,8]. These biofilms protect the bacterial population from both environmental stressors and antimicrobial agents, increasing resistance by 10- to 1000-fold compared to their planktonic counterparts [2]. The matrix of the biofilm protects the bacteria from antibodies and antimicrobial peptides and reduces the effects of the complement system and phagocytosis. At the same time, to adapt to changes in the environment, *K. pneumoniae* has a system that coordinates the signals and responses that control gene expression in a microbial population [31]. KAC and KoSC carry several fimbrial and non-fimbrial adhesion genes, including *fimH* and *mrkD*. The biofilm regulator gene mrkH is present in over 99% of genomes, while KoSC isolates moderate levels of biofilm in only approximately 78% of isolates [4,5].

### 3.2. Surface Polysaccharides

The polysaccharide capsule (CPS) or K-antigen is a complex polysaccharide layer bound to the surface protein Wzi and is a critical virulence factor of *Klebsiella* spp., shielding the bacteria from host immunity. Polysaccharide capsules act as an antiphagocytic layer, preventing recognition and internalization by macrophages and neutrophils [30]. The capsule modulates complement deposition, including C3b, thereby inhibiting membrane attack complex (MAC) formation and subsequent osmotic lysis [1]. Additionally, the capsule dampens early inflammatory responses by inducing lower levels of proinflammatory cytokines like TNF- α and IL-6 while promoting anti-inflammatory IL-10, allowing the pathogen to establish an infection niche [4,5,8].

Hypermucovisity (HMV) is a phenotypic trait defined by the production of large amounts of capsular material or macromolecular exopolysaccharides, resulting in highly viscous colonies. This multilayer barrier protects against innate and humoral immunity by preventing macrophages and neutrophils from recognizing, binding, and internalizing bacteria. The anionic polysaccharides also act as chemical decoys, binding cationic antimicrobial peptides such as human defensins and lactoferrin before they reach the bacterial membrane [8,17,32].

HMV enables *K. pneumoniae* to shift from a localized to a systemic invasive pathogen [18], crossing mucosal barriers and spreading via the bloodstream even in immunocompetent hosts, producing a “hypervirulent” syndrome [30]. This includes spontaneous pyogenic liver abscess, endophthalmitis, and meningitis, often metastatic infections causing severe morbidity or mortality in otherwise healthy individuals [18]. HMV also promotes biofilm formation, especially on abiotic surfaces such as catheters and internal medical devices [2]. CPS and HMV are related but distinct: CPS forms the structural envelope, whereas HMV is the regulated overproduction of materials driven by the *rmpA*, *rmpA2*, and *rmpD* genes on large virulence plasmids or integrative chromosomal elements [8]. HMV remains a defining trait of hypervirulent *K. pneumoniae*. According to current research of 588 KoSC genomes, no HMV genes like *rmpA* and *rmpA2* were found within the complex [27]. Additionally, in KAC, large-scale genomic analysis shows no *rmpA* gene, except for ST432, which possessed this gene thanks to the *K. pneumoniae* plasmid-borne genetic information it possessed [5].

Recent research highlights three major shifts in *Klebsiella* virulence factors: lesser importance placed on the “string test”, convergence of different pathotypes, and discovery of non-encapsulated phenotypes. Historically, *K. pneumoniae* was divided into two strains, namely crKp and hvKP, where hvKp was defined by hypermucosity [7]. Clinically, hypermucoviscous pathogens are identified using the “string test”, in which lifting a colony from an agar plate produces a viscous string greater than 5 mm [33]. The “string test” is no longer a reliable hvKp marker, as many hypermucoviscous strains lack actual hypervirulence, and some hypervirulent strains are not mucoid. Current research supports a genomic definition of hypervirulence based on five virulence genes: *iucA*, *iroB*, *peg-24*, *rmpA*, and *rmpA2* [18].

Lipopolysaccharide (LPS) or endotoxins are a fundamental structural component and a crucial virulence factor of *Klebsiella* spp. [31]. It supports pathogen survival under immune pressure, facilitates tissue-specific fitness, and mediates resistance to last-resort antibiotics. LPS consists of Lipid A, core oligosaccharides, and O-antigen. Lipid A is the hydrophobic anchor in the outer membrane (OM), a potent pathogen-associated molecular pattern (PAMP), and the main TLR4 ligand, which triggers the release of proinflammatory cytokines and the recruitment of neutrophils and macrophages. Core oligosaccharides link Lipid A to the O-antigen and help maintain outer membrane integrity. The O-antigen is the highly variable outer polysaccharide, with at least nine distinct serotypes in *K. pneumoniae* and seven in KoSC, all of which were previously identified in *K. pneumoniae*, suggesting a shared evolutionary history or frequent horizontal exchange of surface antigen genes [27]. *Klebsiella* can mask LPS from immune detection. In *K. pneumoniae* serotypes such as K1, K10, and K16, a thick capsule shields LPS from TLR4 recognition, and structural modifications of the Lipid A structure can prevent recognition by immune receptors, blunting early inflammatory responses [8,22].

Recent studies have identified non-encapsulated *K. pneumoniae* (NEKp) in clinical specimens, which is paradoxical because CPS is considered to be the main virulence factor for survival and pathogenicity. These bacteria survive via mutations in initial glycosyltransferase genes (e.g., *wbaP* or *wcaJ*) that redirect the undecaprenyl phosphate pool to O-antigen synthesis. Despite CPS loss, excess O-antigen increases biofilm formation and serum resistance. Without CPS, NEKp can evade antibody-mediated killing targeting specific K-serotypes while retaining a strong lipopolysaccharide barrier. These strains disseminate less but persist longer in the environment and host. In the ST11-KL64 sublineage that has replaced ST11-KL47, O-antigen expression faces a scarcity of protective antibodies in human B cells, further enhancing pathogenicity [20].

### 3.3. Siderophore-Mediated Iron Acquisition

Siderophore-mediated iron acquisition is a mechanism of bacterial pathogenesis [1]. Free iron is limited within the human host due to its isolation by proteins (transferrin, ferritin). That is why pathogens use high-affinity chelating agents, called siderophores, to recruit iron for their survival, growth, and replication [8]. Current research identifies four main siderophore systems used by *Klebsiella* genus: enterobactin, yersiniabactin, salmochelin, and aerobactin. Enterobactin is the “basic” iron-uptake system found in nearly all isolates of the *Klebsiella* genus. It has a high affinity for iron but is often neutralized by the human protein lipocalin-2 (siderocalin) secreted by neutrophils during a respiratory infection.

#### 3.3.1. *Klebsiella pneumoniae*

Yersiniabactin (ybt), located on the chromosomal integrative and conjugative elements, evades lipocalin-2 and is a marker of the transition from asymptomatic colonization to active infection. Its prevalence varies in *K. pneumoniae* from 18 in crKp to 90% in hvKp. Salmochelin(iro) produces a C-glucosylated form of enterobactin and is not recognized by the hosts lipocalin-2. Therefore, it can maintain iron acquisition and remain unnoticed by the host’s immune system. Similarly to yersiniobactin, its prevalence in the case of hypervirulent *K. pneumoniae* is >90%, acting as a hypervirulence marker. Aerobactin (iuc) is the most critical determinant of systemic infection and hypervirulence, specifically in hvKp [8]. It has a lower affinity for iron but is highly efficient in promoting growth in human blood and has a 90% activity in hypervirulent strains. This is now recognized as an accurate biomarker for predicting the hypervirulent phenotype in hvKp. These siderophore systems are markers of great clinical risk in *K. pneumoniae* and are now used to differentiate hvKp using the presence of the aerobactin and salmochelin genes, with a diagnostic accuracy of 95% [33].

In the past, high-affinity siderophore systems were found only in antibiotic-susceptible hvKp strains, while crKp strains lacked these enhanced iron-gathering tools. However, because of the evolutionary changes in *Klebsiella* spp., iron acquisition systems have integrated into strains with less developed siderophore systems. Firstly, convergent strains facilitate MDR lineages in acquiring virulence plasmids carrying *iuc* and *iro* operons, or hypervirulent lineages acquiring carbapenemase-encoding plasmids, creating a high-risk strain [26]. Another change is the chromosomal integration of virulence fragments. Iron acquisition genes were once thought to be confined to large, non-conjugative plasmids [8], but research has revealed a new pathway in which virulence plasmid fragments (carrying *iucABCD-iutA* and *rmpA2* clusters) integrate into the chromosome via IS elements such as IS26 and ISKpn1. This integration stabilizes virulence traits and ensures inheritance by all offspring without the metabolic cost of maintaining a large plasmid [34]. Another example of these evolutionary mechanisms is adaptive genetic deletion. Some clones lose specific iron acquisition genes during evolution, which promotes virulence plasmid mobilization and strengthens antioxidant defenses in host macrophages, improving survival. In China, for instance, the dominant ST11-KL64 subclone has lost the *iroBCDN* (salmochelin) cluster. This streamlined virulence profile may facilitate global dissemination [35].

#### 3.3.2. *Klebsiella aerogenes* and *oxytoca* Complex

Recent studies have also identified hypervirulence-associated iron acquisition clusters not only in *K. pneumoniae* but also in other species members, such as KAC, indicating that the threat of enhanced iron acquisition is spreading throughout the genus [5].

The prevalence of yersiniobactin varies depending on the species and strain, ranging from 50 to 100% in KoSC species and from 20 to 48% of KAC isolates. In *K. aerogenes*, it is strictly associated with the expansion of the global pandemic clones ST93 and ST4, and in KoSC, it has been determined in 100% bloodstream isolates in specific region studies marking it as a virulence factor for high-risk lineages also in KAC. In both complexes, the ybt locus is typically carried on ICEKp (integrative conjugative elements of *Klebsiella pneumoniae*) platforms [8,26,27].

In KAC, the salmochelin operon is found in 92% of genomes, but in an incomplete state, representing its use as an ecological fitness factor, enhancing colonization and survival within environmental niches, rather than virulence driver. The salmocehlin system, represented by the iroN receptor in KoSC, is present in 41,3% of the complex, mostly in *K. michiganensis* (75,9%), and much less frequent in other species, like *K. oxytoca* (21,3%) and *K. pasteurii* (0%) [8,36,37].

In KoSC and KAC species, the complete aerobactin operon is extremely rare, but there is a nearly ubiquitous presence of the aerobactin receptor iutA, which indicates scavenging of iron-siderophore complexes produced by other bacteria in a polymicrobial environment. Additionally, in KAC, it is used for ecological functions other than iron uptake, such as acting as a receptor for cloacins (bacteriocins), which provide an advantage in inter-bacterial competition. A notable critical evolutionary event is the clinical isolate of *Klebsiella aerogenes* ST432 carrying the complete *iucABCD-iutA* operon on a massive hybrid plasmid, likely acquired via interspecies horizontal gene transfer from *K. pneumoniae*, representing a new hypervirulence evolution in KAC. Additionally, emerging high-risk KoSC lineages like ST199 and ST2 are increasingly acquiring similar hybrid plasmids that co-carry aerobactin variants and carbapenemases, driving the evolution of more virulent, iron-scavenging lineages [5,17,28,36].

### 3.4. Toxins and Secretion Systems

Toxins and secretion systems are key offensive tools for bacteria. While capsules and siderophores mainly support defense and nutrient uptake, toxins directly damage host cells, and secretion systems export these toxins or exchange genetic material. In *Klebsiella*, toxins include endotoxins such as LPS, which trigger proinflammatory cytokine release, and genotoxins that damage host DNA. A major focus of research has been colibactin, a genotoxin encoded by the pks genomic island on integrative and conjugative chromosomal elements. Colibactin is a polyketide-peptide that causes double-strand DNA breaks, chromosomal aberrations, and cell cycle arrests in host cells and is found on ICEKp in both KpSC and KAC. Frequently carried toxins by KAC strains are bacteriocins used in inter-bacterial competition. These toxins (cloacins/colicin E3) inhibit the growth of closely related bacterial strains, allowing them to eliminate their environmental competitors. A similar role in establishing environmental dominance is possessed by microcin E492 in hvKp [8,36].

In KoSC, the cytotoxins tilivalline and tilimycin are tricyclic pyrrolobenzodiazepine (PBD) metabolites produced via bimodular non-ribosomal peptide synthetase (NRPS) pathways. Tilivaline binds to tubulin and induces mitotic arrest, whereas tilimycin is a potent genotoxin that induces double-strand DNA damage. These toxins are the primary drivers of pathological mucosal hemorrhage in AAHC following antibiotic-induced gut dysbiosis [4].

Secretion systems are complex protein machineries in the bacterial cell envelope, classified as type I to type XI [38]. They deliver effector proteins to the extracellular environment, host cells, or transfer DNA between bacteria during conjugation [39]. Based on protein composition, evolutionary origin, and transport mechanisms, secretion systems are categorized into distinct types. According to current research, *Klebsiella* utilizes four secretion systems (type I, II, IV, VI). The type I secretion system (T1SS) exports toxins, antibiotics, digestive enzymes, and repeat toxin proteins into the extracellular milieu, enhancing *K. pneumoniae* adaptability and pathogenicity. Strains with higher hypervirulence have a greater prevalence of T1SS. The type II secretion system (T2SS) secretes pullulanase, which breaks down carbohydrates, and ADP-ribosylating toxins, contributing to host tissue damage [40]. As portrayed in Table 2, among various virulence factors, the type IV secretion system (T4SS) is the primary mechanism for bacterial conjugation and a key driver of *Klebsiella* evolution [16]. It mediates the horizontal transfer of plasmids carrying carbapenemases and virulence factors. While traditional hypervirulence plasmids were non-conjugative, recent research has identified “fusion” or “superplasmids” with integrated T4SS clusters, allowing for the simultaneous transmission of hypervirulence and MDR [22]. The type VI secretion system (T6SS), involved in inter-bacterial competition and virulence, allows *Klebsiella* to inject toxic effectors into rival bacteria, facilitating niche dominance, and into host cells, ensuring virulence [3]. KpSC strains often carry three distinct T6SS clusters (I-III). A study in Armenia found that ST307 isolates carried nearly entire sets of all three clusters [41]. *Klebsiella aerogenes* possesses a unique type of i3 T6SS (most other species have only type i2), which aids its survival in nosocomial environments by eliminating competitor bacteria and resisting other microbial flora. It serves as a “molecular syringe” for the delivery of toxic effectors into the cytoplasm of other cells. Combined with its unique flagellar motility, *K. aerogenes* effectively colonize antibiotic-rich hospital niches [5].

Other secretion systems that transport macromolecules other than proteins are not included in the numbered classification but are essential for the assembly of the previously discussed virulence factors. The chaperone–usher pathway assembles and stabilizes subunits of protein into a polymeric structure anchored to the bacterial surface (as opposed to protein secretion systems that release them into the extracellular space), creating fimbriae (Type 1 fimbriae; Type 3 fimbriae) [38]. Finally, the Wzx/Wzy pathway, although technically an export pathway for capsular polysaccharides and macromolecular exopolysaccharides, functions as a “secretion system” essential for the hypermucoviscous phenotype. Wzx acts as a flippase, while Wzy polymerizes the sugar moiety, providing a physical shield against phagocytosis and complement-mediated killing [8].

## 4. Antimicrobial Resistance

AMR represents a critical shift in clinical microbiology from isolated resistance phenotypes to MDR, XDR, and PDR pathogenicity [29]. AMR evolution in *Klebsiella* is driven by high genetic plasticity and selective pressure from the widespread misuse of broad-spectrum antibiotics [42], which is a pattern seen globally. For example, isolates from Ukrainian war victims revealed extreme resistance, with several strains classified as pandrug-resistant. These isolates (ST23, ST147, ST395, ST512) are often hypervirulent and resistant to all tested antibiotics, including carbapenems, cephalosporins, and colistin [10]. This evolution proceeds through three primary mechanisms: clonal expansion and replacement, horizontal gene transfer/acquired mobile genetic elements, and adaptive point mutations.

Horizontal gene transfer accounts for most acquired resistance through large conjugative plasmids and other mobile genetic elements, including transposons and integrons, which accelerate the spread of antibiotic resistance genes [43]. These plasmids carry carbapenemase and extended-spectrum β-lactamase (ESBLs) genes between strains and even across species. *K. pneumoniae* is the most common species in clinical practice, representing about 86% of isolates, and it is the main driver of carbapenem-resistant outbreaks via carbapenemases (*KPC*, *NDM*, *OXA-48-like*). In China, the *blaKPC-2* gene occurs in about 89.4% of crKp isolates [20], while NDM-type enzymes are more prevalent in the Indian subcontinent and parts of Europe [7,21]. Acquired carbapenemase genes are found in approximately 18.8% of KAC genomes, with the most prevalent enzymes being KPC-2/-3 associated with Tn4401 and IS26 elements. KoSC species also increase their AMR through *blaKPC-2* carrying plasmids like, IncN, or IncF. Other plasmid-borne resistance genes are *KAC*-acquired polymyxin and colistin resistance through plasmid-borne *mcr* genes and KoSC-acquired colistin resistance through acquisition of plasmid-borne *mcr-1* or *mcr-9* [4,36].

Mutations at specific bacterial sites can prevent antibiotic binding, rendering them ineffective. In *K. pneumoniae*, alteration in penicillin binding proteins (PBPs) lowers their affinity for β-lactam antibiotics, increasing resistance [4]. Resistance to last-resort antibiotics like colistin and tigecycline often arises from chromosomal mutations in regulatory genes (e.g., *mgrB*, *ramR*) that alter target sites or upregulate protective mechanisms like efflux pumps [4,13,36].

Another resistance mechanism is the alteration of outer membrane (OM) permeability. Antibiotics must first cross the OM to reach their targets. A mutation that deletes or modifies OM porins, such as *OmpK35* and *OmpK36*, limits antibiotic entry into the bacterial cell [36,39]. For instance, the *OmpK36GD* mutation significantly reduces carbapenem susceptibility [26].

Resistance also arises from the overexpression of efflux pumps caused by mutations in the repressors of the AcrAB-TolC and OqxAB multidrug efflux systems (*acrR*, *ramR*). These pumps actively expel quinolones, tigecycline, and some β-lactams, providing broad resistance even in strains lacking specific resistance genes [4,39]. Lastly, resistance to specific antibiotics like colistin is often mediated by LPS modification via *mcr* genes or mutations in the *phoPQ/pmrAB* two-component systems and their negative regulator *mgrB* [4,31,36].

As seen in Table 3, the resistance mechanisms of *Klebsiella* spp. are multifaceted, often involving the synergy of several genetic factors. A key mechanism is enzymatic hydrolysis: the production of β-lactamases, and ESBLs, as well as aminoglycoside-modifying enzymes (AMEs), inactivate antibiotics. For example, ESBLs (e.g., *blaCTX-M-15*) inactivate cephalosporins, while carbapenemases (e.g., *blaKPC*, *blaNDM*, *blaOXA-48*) hydrolyze nearly all available β-lactams [39]. AMEs and 16srRNA methyltransferases (*armA*, *rmtC*) provide aminoglycoside resistance by altering the drug or its ribosomal target [31]. These hydrolyzing genes are often upregulated upon antibiotic exposure, further increasing resistance [42].

The spread of AMR makes these pathogens increasingly difficult to treat. Alarmingly, resistance has emerged even to “last-resort” antibiotics, including *blaNDM-1* for carbapenems, *tet(x4)* for tigecycline, and the *mcr-1* gene for colistin resistance, where colistin resistance generally remains low (<10%), except for specific clones [17,39,44]. Historically, carbapenem was the “gold standard” for treating *Klebsiella*, but their efficacy is now severely compromised by the growing prevalence of KPC- and NDM-producing strains in KpSC, KAC, and KoSC [4,5,13]. Ceftazidime–avibactam (CZA) is a novel combination effective against KPC-producing strains but not against metallo-β-lactamases (MBLs) like NDM. Polymyxins and tigecycline are last-resort options, but their use is limited by high nephrotoxicity and increasing resistance mediated by *mcr* genes or efflux pumps [39]. Tigecycline resistance is an emerging threat in KoSC contributed by plasmid-mediated tet(X4) monooxygenases in lineages like ST534 and ST3393 [17].

To manage these infections, different resistance pathways are being investigated. W. Jiang et al. showed that using Zinc Finger Nuclease to upregulate PBPs and downregulate ESBL production increased susceptibility to several antibiotics [42]. Other studies indicate that traditional Chinese medicine derivatives, such as berberine hydrochloride and nordihydroguaiaretic acid (NDGA), can inhibit crKp growth or act as efflux pump inhibitors, resensitizing bacteria to agents like ceftriaxone [2].

Therefore, future treatment strategies should focus not on expanding antibacterial agents but on alternative methods for resistant bacteria [45]. One of these methods is phage therapy, where commercial and pre-adapted phage cocktails have shown strong in vitro activity against XDR and PDR clinical isolates, suggesting their use as an alternative or adjunct to antimicrobials. Another option is Chinese medicinal compounds: in vitro studies of berberine hydrochloride show antibacterial activity against crKp isolates, indicating potential clinical value [46]. Additionally, immunological strategies, including vaccines and monoclonal antibodies targeting conserved surface polysaccharides (K- and O-antigens), aim to protect against many serotypes [1]. Finally, molecular targeting studies seek new inhibitors of specific virulence factors, such as aerobactin synthesis, to attenuate hypervirulent strains, and lower mortality [47].

## 5. Conclusions

*Klebsiella* spp. are central to the global antimicrobial resistance (AMR) crisis. This article highlights the worldwide emergence of “superbugs” that combine hypervirulence and multidrug resistance due to striking genetic plasticity and the growing convergence of diverse strains. Two main evolutionary routes have been identified, namely carbapenem-resistant hypervirulent *K. pneumoniae* and hypervirulent carbapenem-resistant *K. pneumoniae*, representing distinct paths to the same convergence of resistance and hypervirulence.

An increasing volume of virulence and resistance information is being mobilized through chromosomal insertions, with T4SS emerging as a key driver of both virulence and resistance gene dissemination. *K. pneumoniae*, responsible for an estimated 20% of AMR-attributable deaths and listed by the WHO as a “critical priority pathogen”, is the primary focus of current research. It causes a wide range of infections, including pneumonia, bacteremia, meningitis, and pyogenic liver abscess. The simultaneous rise in resistance and virulence greatly complicates clinical management.

The systematic study of virulence determinants and antimicrobial resistance mechanisms in *Klebsiella* can unlock powerful tools for prevention, rapid diagnosis, and targeted therapy. Clarifying the molecular basis of these traits is crucial for better prevention, surveillance, and treatment strategies. Although *K. pneumoniae* is the most clinically important species, others such as *K. oxytoca*, *K. aerogenes*, and *K. variicola* are also showing increased virulence and resistance and often act as reservoirs for high-risk resistance plasmids and other mobile elements. Because research and surveillance focus mainly on *K. pneumoniae*, non-pneumoniae *Klebsiella* are under-monitored, and the true burden of hypervirulent and MDR strains is likely underestimated. An expanded study of virulence and resistance in these species is urgently needed.

There is a pressing need for rapid genome-based diagnostics to replace or supplement slow, insensitive, and nonspecific phenotypic assays. Future work should adopt a One Health approach, examining how environmental reservoirs (wastewater, surface water, soil, livestock) serve as hubs for the horizontal transfer of resistance and virulence genes among humans, animals, and the environment. The misuse of antibacterials in healthcare, agriculture, and pharmaceutical production is a major driver of AMR in *Klebsiella* and other bacteria.

The world risks a “clinical crisis” in which *Klebsiella* spp. become resistant to all available drugs, including last-resort agents like colistin, underscoring the need to prioritize non-traditional therapies. Promising options include bacteriophage therapy, monoclonal antibodies, and small-molecule inhibitors targeting virulence factors such as aerobactin and other siderophore-mediated iron acquisition systems. Combining these innovative treatments with strong infection prevention, antimicrobial stewardship, and One Health-based surveillance will be essential to control hypervirulent MDR *Klebsiella* spp.

## Figures and Tables

**Table 1:** Geographical epidemiology of *Klebsiella* species and sequence types.

Species	Sequence Types (ST)	Geographical Distribution and Epidemiological Context
*K. pneumoniae*	ST11	Predominantly in China and Asia (also detected in America and Europe)
	ST258/ST512	ST258 is the dominant CRKP in the USA and Israel ST512 (ST258 variant) found in Italy and Southern Europe
	ST147	The UK, India, Bangladesh, Greece, Mediterranean, Italy
	ST23	Asian Pacific Rim (Taiwan, China). Emerging in Europe (Ireland, France, Finland, Sweden) and the USA
	ST307	The USA, Italy, Colombia, Korea, Pakistan, South Africa
	ST395	France, Russia, Germany, Armenia
	ST15	China, the UK, Europe, Vietnam
	ST101	Europe (Italy, Serbia, Romania, the UK)
	ST231	South Asia (India, Bangladesh), Thailand
	ST65/ST86	ST65 in East Asia ST86 in Australia, Canada, Caribbean
*K. oxytoca* complex	ST2, ST9	ST2 in Germany, the UK, and Ireland ST9 Japan
	ST4	Austria
	ST179	Norway
	ST220	Tunisia
	ST199	The US
	ST176	Worldwide (Australia, Sweden, the UK)
*K. aerogenes*	ST93/ST4	Worldwide (North America, Europe, Asia)
	ST432	The USA
	ST14, ST208	Western China
*K. variicola*	ST771	Bangladesh

**Table 2:** Virulence factors and genetic determinants of *Klebsiella* genus.

Virulence Factor	Associated Genes	Biological Function	Pathogenic Effect
Capsular Polysaccharide (CPS)	*wzi*, *wza*, *wzb*, *wzc*, *gbd*, *wca*, *galF*, *ugd*, *wbaP*, *wcaJ*	Forms a physical barrier, inhibits C3 complement deposition and phagocytosis	Primary virulence determinant, immune stealth and serum resistance
Mucoid Regulators (Hypermucovisity)	*rmpA*, *rmpA2*, *rmpD*	Regulate *cps* operons to overproduce capsule and exopolysaccharides	Hypervirulence Hypermucoviscous phenotype, systemic dissemination and invasive syndromes
Aerobactin (Siderophore)	*iucABCD*, *iutA*	High-affinity iron acquisition; dominant siderophore in hvKp	Key biomarker for hypervirulence
Salmochelin (Siderophore)	*iroBCDN*, *iroE*	C-glycosylated enterobactin that evades lipocalin-2	Colonization, persistence in host
Yersiniabactin (Siderophore)	*ybtS*, *ybtAEPQSTUX*, *irp1*, *irp2*, *fyuA*	Iron sequestration	Virulence/Adaptation: Respiratory tract infection and dissemination from the lungs
Enterobactin (Siderophore)	*entABCDEF*, *fepABCDG*	Highest-affinity iron uptake system	Basic growth, neutralized by host lipocalin-2
Lipopolysaccharide (LPS)	*wb*, *waa*, *lpx*, *uge*, *wabG*	Endotoxin; immunity to host immune system	Potent immune activator (TLR4 ligand); avoids complement-mediated lysis
Type 3 Fimbriae (Pili)	*mrkABCDF*, *mrkH*, *mrkJ*	Adhesion	Resistance/Persistence: Robust biofilm formation on medical devices
Type 1 Fimbriae (Pili)	*fimABCDEFGHIK*, *fimK*	Adhesion	Colonization of the urinary tract
Colibactin (Genotoxin)	*ClbA-S*, *pks* genomic island	Double-strand DNA breaks, cell cycle arrest in host cells	Hypervirulence: Meningeal tropism, colonization persistence
PBD Cytotoxins (KoSC-specific)	*npsA*, *npsB*, *aroX*, *adsX*, *hmoX*	Tilivalline and tilimycin production, mitotic arrest and DNA damage	Cause for AAHC
Type VI Secretion System (T6SS)	*tssF*, *tssG*, *tle1*, *tli1*	Toxic effector delivery	Inter-bacterial competition
Type IV Secretion System (T4SS)	*tra operon (traA*, *traB*, *traU*, *traV*, *traY)*, *virB cluster (virB1–11)*	Mediates bacterial conjugation and horizontal gene transfer, plasmid mobilization	Pathotype convergence
Metabolite Transporter	*peg-344*	Inner membrane transporter for unidentified growth factors	Biomarker for hypervirulent phenotype
Allantoin Metabolism	*allS*, all gene cluster	Allantoin utilization as a carbon and nitrogen source	Liver-invasive syndrome adaptation
Flagellar Motility (*K. aerogenes* only)	*flhD*, *fliA*, *flgL*, *fliR*, *fliE*, *fliT*	Flagellar assembly, bacterial chemotaxis	Motility

**Table 3:** Comparative clinical and resistance profiles of *Klebsiella* Species.

	*K. pneumoniae*	*K. oxytoca* (Complex)	*K. aerogenes*
Clinical manifestation	Pneumonia, urinary tract infections (UTIs), bloodstream infections (BSIs), pyogenic liver abscess, meningitis, endophthalmitis, necrotizing fasciitis	Pathognomonic antibiotic-associated hemorrhagic colitis (AAHC), UTI (high prevalence in pregnancy), BSI, hospital-acquired pneumonia (HAP), peritonitis, skin and soft tissue infection, meningitis	Nosocomial respiratory tract infections, primary and secondary BSIs, UTI, meningitis, surgical site infections
Dominant resistance mechanisms	Enzymatic: Carbapenemases (KPC, NDM, OXA-48, VIM, IMP) and ESBLs (*blaCTX-M-15*, SHV, TEM) Structural: Porin loss or modification (*OmpK35*, *OmpK36*) Efflux pumps: Overexpression of AcrAB-TolC and OqxAB systems Target site modification: PBP structural alterations, LPS modification (*mgrB*, *phoPQ*, *pmrAB*) for colistin, QRDR mutation (*gyrA*, *parC*) for quinolones	Intrinsic: Chromosomally encoded *blaOXY* (OXY-1 to OXY-12) encoding resistance to amino-and carboxypenicillins Acquired: Plasmid-mediated carbapenemases (KPC-2, NDM-1, OXA-48, VIM-1) Target/Efflux: *mgrB* mutations (*mcr-1/9*) colistin resistance genes, and OqxAB pumps	Intrinsic: Chromosomally located *ampC* gene (AER-1 to AER-196) conferring resistance to β-lactams except cefepime and carbapenems Acquired: Transfer of carbapenemases (KPC-2, NDM, OXA-48), ESBLs (CTX-M-15, SHV-12) and *mcr* genes (*mcr-1*, *-9*, *-10*) Efflux/Regulatory: Open genome facilitating accumulation of AcrAB-TolC pumps

## Data Availability

No new data were created or analysed in this study. Data sharing is not applicable to this article.

## Abbreviations

KpSC	*Klebsiella pneumoniae* species complex
KoSC	*K. oxytoca* species complex
AAHC	Antibiotic-associated hemorrhagic colitis
cKp	Classical *K. pneumoniae*
hvKp	Hypervirulent *K. pneumoniae*
MDR	Multidrug resistance
AMR	Antimicrobial resistance
XDR	Extensively drug-resistant
PDR	Pandrug-resistant
crKp	Carbapenem-resistant *K. pneumoniae*
ISs	Insertion sequences
KAC	*K. aerogenes* complex
ICU	Intensive care unit
USA	United States of America
UK	United Kingdom
CZA	Ceftazidime–avibactam
CPS	Polysaccharide capsule
MAC	Membrane attack complex
HMV	Hypermucovisity
LPS	Lipopolysaccharide
OM	Outer membrane
PAMP	Pathogen-associated molecular pattern
NEKp	Non-encapsulated *K. pneumoniae*
T4SS	Type IV secretion system
T6SS	Type VI secretion system
PBPs	Penicillin binding proteins
AMEs	Aminoglycoside-modifying enzymes
